# Using visual methods to further enhance qualitative evidence synthesis

**DOI:** 10.1002/cesm.70009

**Published:** 2024-12-22

**Authors:** Mayara Silveira Bianchim, Emma F. France, Jane Noyes

**Affiliations:** ^1^ School of Medical and Health Sciences Bangor University Bangor UK; ^2^ Centre for Healthcare and Community Research, Faculty of Health Sciences and Sport University of Stirling Stirling UK

**Keywords:** meta‐ethnography, QES, qualitative evidence synthesis, visual methods

## Abstract

**Background:**

The use of visual methods in qualitative evidence synthesis (QES) adds a valuable dimension to the synthesis process by enhancing understanding and knowledge generation. Visual methods are currently underused and underreported in QES.

**Methods:**

This is the first study to describe accessible visual methods that support various stages of QES and to show the application of visual methods to a Cochrane QES of 43 studies using meta‐ethnography and systematic review methods. This study also addresses the involvement of stakeholders including the public, practical considerations of equity, diversity, inclusion, and reflexivity in the selection and application of visual methods.

**Results:**

In a novel approach, the review authors utilized a combination of remote and in‐person visual methods to initiate and develop their synthesis, involving stakeholders throughout the process. The review authors used methods including paper labels, cartoons, infographics, virtual whiteboards, and diagrams. The rigorous use of visual methods in the QES facilitated data visualization, remote analysis meetings, interpretation of extensive data, and meaningful patient and public involvement.

**Conclusion:**

QES authors are encouraged to consider the use of visual methods, particularly when involving the public in the synthesis process. When selecting visual methods, authors should consider how they align with the study's objectives, suit the stage of synthesis, might enhance analysis, their available resources, and the team's technical skills.

## Plain Language Summary

Using visual methods in qualitative evidence synthesis (QES) enhances transparency in the process, helping to understand and create new knowledge. This paper gives an overview of accessible visual methods used at different stages of a QES, with examples from a Cochrane QES that used meta‐ethnography. The importance of including stakeholders, and considering equity, diversity, inclusion, and reflexivity when choosing and using these methods are also outlined. A worked example of a QES using meta‐ethnography shows how visual methods can support synthesis. The authors used a mix of remote and in‐person visual methods to start and develop their synthesis, involving stakeholders throughout. These methods included paper labels, cartoons, infographics, virtual whiteboards, and diagrams. Visual methods helped with data visualization, remote group meetings for analysis, understanding large amounts of data, and meaningful involvement of patients and the public. QES authors are encouraged to use available visual methods, especially when involving the public in the synthesis process.

## 1 BACKGROUND

Visual methods represent a novel approach in qualitative evidence synthesis (QES) by introducing another dimension to the synthesis process and contributing to the understanding and generation of knowledge [1]. The terminology “qualitative data or evidence” broadly refers to findings from primary qualitative studies (e.g., analysis of data from interviews, focus groups, and the production of new theories or theoretical insights), or qualitative data (such as narrative responses to open ended questions). Numerous methods can be used for data synthesis in a QES including meta‐ethnography [2], thematic synthesis [3], and framework synthesis [4]. Irrespective of the method used for analysis and synthesis, additional visual methods can play a crucial role in aiding review authors and readers to comprehend, organize and display qualitative data collected from included studies [5].

There is a tendency for review authors to underutilize the diverse array of visual display methods, tools, and techniques available to enhance a chosen synthesis method [5]. Some review authors may assume that their selected synthesis method includes all necessary supplementary methods, tools, and processes, while others may lack the skills or confidence to generate alternative formats for their synthesis. This paper provides an overview of accessible visual methods supporting the synthesis stages of a QES, with examples and their application in the development of a Cochrane QES using meta‐ethnography. The paper also addresses the role of stakeholders, considerations of equity, diversity, and inclusion, and reflexivity in selecting and employing additional visual methods. Although the focus is on QES, the visual methods described here can also be used to facilitate communication of complex and sensitive topics in primary qualitative research during data collection and analysis [6, 7, 8].

### 1.1 Overview of visual methods and their uses

Table 1 presents an overview of visual methods according to their role in the synthesis process (i.e., support and develop synthesis, facilitate stakeholder engagement, and record the synthesis process). Visual methods can be used in different ways at various timepoints to support the synthesis of qualitative evidence, such as data display and management, initial exploration of data, and synthesis. Visual methods can help with the development of new insights from data allowing a deeper understanding and construction of new knowledge [9].

**Table 1:** Visual methods to support review planning, conduct, and synthesis.

Category	Visual method	Format available	Use	Strengths and limitations
Support and develop synthesis	Flipcharts [10]	Physical (paper, pens of different colors) Virtual (Padlet) [11]	Can be used at any stage to help with ideas development, concept clarification, and linking data and ideas	**Strengths** Easy and cheap to use
				**Limitations** Needs a facilitator and can only accommodate small scale and discrete clarification tasks. Paper version needs to be photographed for easy storage
	Labels [12, 13]	Physical (paper e.g., colored post it notes) [12, 13] Virtual (Padlet, [11] NVivo, [14] EPPI reviewer) [15]	Can be used at initial exploration of data to help with ideas development Particularly useful for linking data	**Strengths** Allow interactive work with multiple researchers face‐to‐face in real time
				**Limitations** Physical version is more suitable to initial exploratory analysis. It might not allow in‐depth analysis of data. Paper version can be time consuming to prepare and needs to be photographed for easy storage. Virtual version allows in‐depth analysis but it might not allow interactive work with multiple researchers face‐to‐face in real time.
	Diagrams [16, 17, 18]	Physical (paper) [12, 13] Virtual (Microsoft Whiteboard) [19]	Can be used at any stage to help with ideas development and concept clarification. Particularly useful for linking data. Causal loop diagrams are an approach to understanding and visualizing systems and share parallels with logic models.	**Strengths** Facilitates collaboration of multiple researchers face‐to‐face or virtually. Useful for small scale and clarification tasks. It can also be used to finalize and express the synthesis.
				**Limitations** Not suitable for identification of themes or in‐depth analysis of data
	Whiteboard [19, 20]	Physical (paper or plastic), pens of different colors Virtual (Google Jamboard) [21]	Can be used at any stage to help with ideas development and concept clarification. Particularly useful for linking data as it enables the creation of large conceptual maps.	**Strengths** The virtual version allows interactive work in real time. Allow analysis of large amounts of data under different themes and categories. It is easy and cheap to use.
				**Limitations** Better suited for a small team of researchers. The paper version needs a facilitator and accommodates smaller scale analysis in comparison to the virtual whiteboard. Paper/plastic version needs to be photographed for easy storage.
	Logic models [22]	Physical (paper) [12, 13] Virtual (Microsoft Visio) [23]	Logic models can be used to show a program theory of how an intervention or system works. Can be used and further evolved at all review stages.	**Strengths** Facilitates the analysis of complex interventions and programs
				**Limitations** Can be mechanistic and might not always represent the dynamic and nonlinear nature of complex interventions
Facilitate stakeholder engagement	Cartoons [12, 13, 24]	Physical (paper) Virtual (virtual storyboard) [18]	Helpful with ideas development and concept clarification. Particularly useful for stakeholder involvement and engagement.	**Strengths** Allows accessible real‐time interactive work. Helpful to display findings in an engaging and simple way, useful to engage with key stakeholders and patient and public representatives.
				**Limitations** Needs a facilitator and it is time consuming
	PowerPoint slides [12, 13]	Physical (printed on paper) Virtual (Microsoft Office PowerPoint) [25]	Presentation of ideas and options for discussion	**Strengths** Easy and cheap to use. It can be useful to engage with key stakeholders and patient and public representatives.
				Limitations Needs a facilitator
	Drawing [12, 13, 18]	Physical (paper) Virtual (Adobe Fresco) [26]	Can be used at any stage to help with ideas development, concept clarification, and linking data and ideas. Also helpful in conveying findings. Particularly useful for stakeholder engagement and involvement.	**Strengths** Works as a visual aid and can be a helpful during interpretation of overarching storyline
				**Limitations** Not instrumental to data analysis. The artists who are not knowledgeable of the topic may misrepresent what is said. Paper/plastic version needs to be photographed for easy storage.
	Collage [27]	Physical (real‐time creation of a collage on paper or whiteboard) Virtual (Canva) [28]	Can be used at any stage to help with ideas development, concept clarification, and linking data and ideas. The collage brings together ideas and interpretations in a visual format that can tell a story in an accessible way.	**Strengths** Can help create positive, accessible and real time engagement and dialog with key stakeholders and patient and public representatives
				**Limitations** Expensive and time consuming. The artists who are not knowledgeable of the topic may misrepresent what is said, but the collage can be rubbed out and corrected. Paper/plastic version needs to be photographed for easy storage.
	Infographic [29]	Physical (paper) Virtual (Canva, Visme [28, 30]	Used after idea or concept is developed but it can help with clarification of concepts and linking data and ideas. The infographic brings together ideas and interpretations in a visual format that can tell the key components of a story in an accessible way.	**Strengths** Can help create positive, accessible engagement and dialog with key stakeholders and patient and public representatives
				**Limitations** Expensive and time consuming. Requires skills or software for production. Paper version needs to be photographed for easy storage.
	Performance [31]	Physical (facilitator or actors telling a story)	Can be used at any stage to help with ideas development, concept clarification, and linking data and ideas. Particularly useful for stakeholder engagement and involvement.	**Strengths** Allows accessible real‐time interactive work. Helpful to display findings in an engaging and simple way, useful to engage with key stakeholders and patient and public representatives.
				**Limitations** Needs a facilitator and it is time consuming
Recording synthesis process	Video [12, 13]	Physical (Digital camera) Virtual (Microsoft Teams or Zoom) [32, 33]	Helpful for storage and organization of visual data	**Strengths** Works as a visual aid and can help to record analysis and manage data
				**Limitations** Not instrumental to data analysis
	Photography [12, 13, 18]	Physical (Digital camera) Virtual (Print Screen)	Helpful for storage and organization of visual data. Can be useful for stakeholder engagement and involvement.	**Strengths** Works as a visual aid and it is helpful to record analytical processes done on paper (e.g., configuration of data using paper labels)
				**Limitations** Not instrumental to data analysis

### 1.2 Considerations when selecting a visual method to support the synthesis

Visual methods should be selected based on whether they can add value to facilitating the synthesis or be used as an integral part of the analytical process (e.g., use of diagrams to develop and visualize the synthesis). All of the visual methods listed in Table 1 are flexible and adaptable to different tasks and the creativity of the review team.

When selecting visual methods, it is important to first consider the content and objectives of the synthesis. The choice of method should align with the analytical goals, such as enhancing understanding or encouraging collaboration within the research team. For example, some visual methods may be more effective for developing early‐stage ideas, while others might help to present findings more clearly.

Delivery considerations, particularly when working remotely, also play a key role. Resources such as internet connectivity, relevant hardware (e.g., computers), and software (e.g., Microsoft Teams) [33] are essential if implementing visual methods online. Remote working also demands technical knowledge from the research team. In addition, visual methods involving a group of researchers (whether in person or remotely) can involve high costs, preparation, and may require a facilitator. Practical considerations, such as preparing materials (e.g., paper labels) and developing a plan for how the method will be used, are crucial for group meetings. If using arts or performance‐based methods, the presence of artists or actors may be required.

### 1.3 How to apply visual methods in qualitative evidence synthesis

To illustrate the use of visual methods to support a synthesis, a worked example of a recent QES using meta‐ethnography is used [12, 13]. Meta‐ethnography is one of the most complex QES methods designed to synthesize mainly rich data from primary qualitative studies in a series of steps to develop new theoretical insights and theory [34]. The review authors investigated how children and young people with chronic noncancer pain and their families experience and understand their condition, pain services and treatments [12, 13]. The whole team was involved in conducting the analytic synthesis with two members leading on and carrying out the majority ofthe analytic synthesis. They produced three lines of argument, a model and a theory of chronic pain management. The combination of their lines of argument was named “The journey of living with chronic pain” which expressed the experiences of children and young people with chronic pain and their families from the onset of chronic pain; their struggle to navigate health services seeking a cure, and to have their needs and expectations met; and the outcome, moving on either to prioritize living well with pain or give up hope [12, 13].

### 1.4 Stakeholder involvement and engagement

The review involved a diverse stakeholder group of health professionals, third sector organizations, policy makers, academics, and the public as well as a patient and public involvement (PPI) group of children and young people with chronic pain aged 8–20 years old and parents from across the United Kingdom. The PPI and stakeholder groups were involved throughout the entire review including making decisions about which studies to include in the synthesis and how to group studies in order to analyze and synthesize them, and the analysis and interpretation of findings from primary studies and of preliminary synthesis findings.

### 1.5 Visual methods to display data

**Figure 1:**
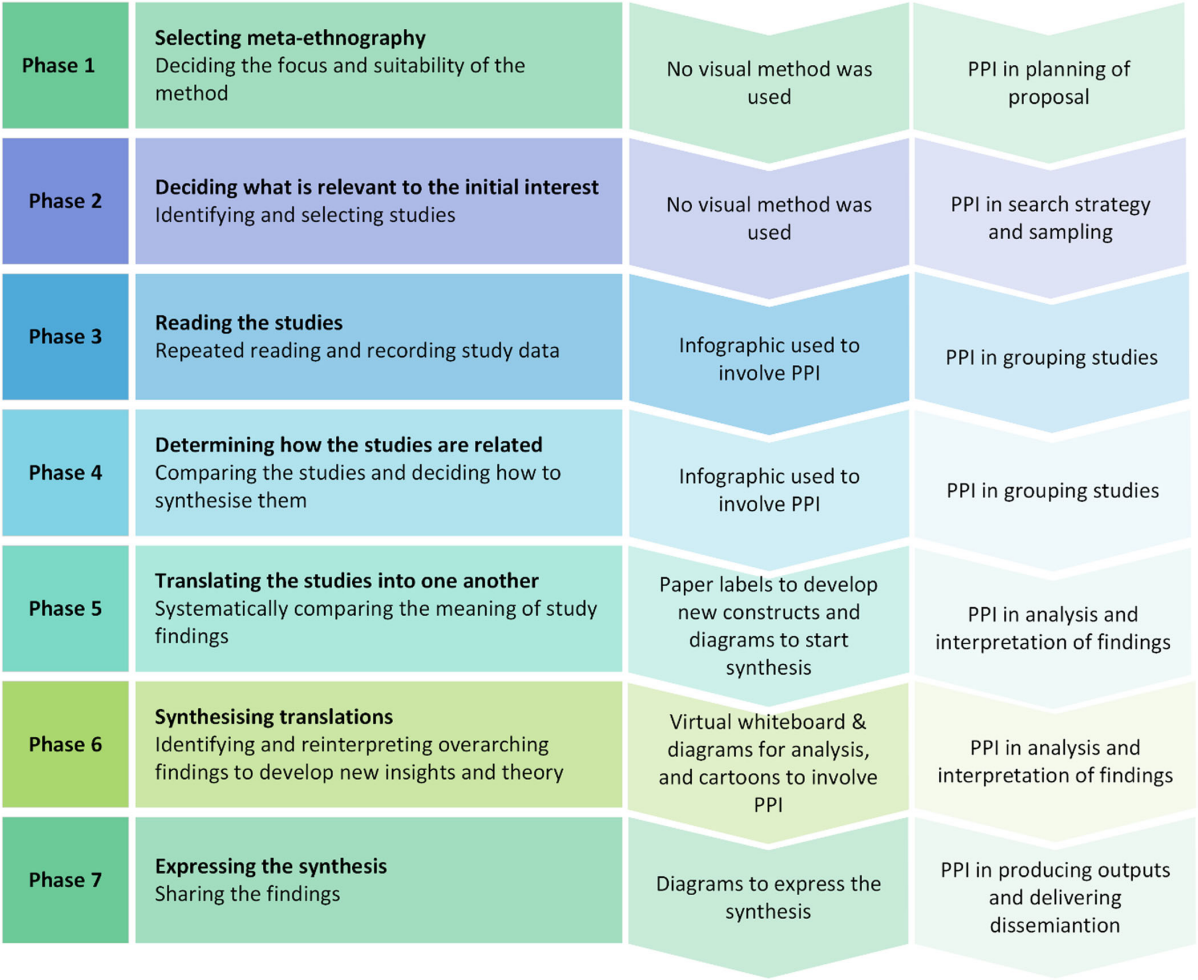
Meta‐ethnography stages and visual methods used at each stage. *Source:* adapted from Noblit and Hare [2]. PPI, patient and public involvement.

France [12, 13] used a variety of visual data display methods (Figure 1) at different stages of their meta‐ethnography to help convey the complex evidence and synthesis, especially to PPIs. The review authors worked mainly remotely as a dispersed team with few opportunities to meet face‐to‐face due to the COVID‐19 pandemic, hence they developed and delivered most of their visual methods virtually. In the absence of guidance on the selection of visual methods, the review authors drew on high‐quality relevant QES reports that used visual display methods successfully [16, 20]. Once the review team gained confidence and found that these visual methods were highly valuable, they selected visual methods to further enhance the synthesis process (Figure 1).

In the following sections, we discuss and evaluate the contribution of each method used in the meta‐ethnography.

### 1.6 Paper labels

**Figure 2:**
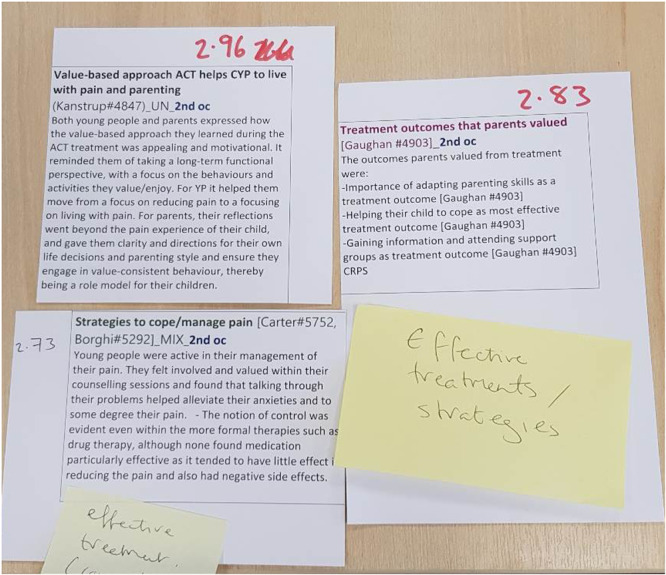
Paper labels used to initiate the process of synthesizing translations during a team meeting.

This method was used among the review team only between phases 5 and 6 as part of the analytic synthesis process (to translate the studies into one another and in synthesizing translations). The review authors used paper labels to initiate the synthesis process and start developing novel insights (Figure 2). In a previous step, at least two review authors interpreted the meaning of every relevant finding, concept, or theme from the studies using NVivo [14] version 12. The authors then compared the meanings within and across studies to identify common or unique concepts. Where possible, the common concepts were then matched, merged, and further interpreted by two review authors and discussed with the wider team of review authors to develop new interpretations. The common concepts and new interpretations were summarized on paper labels. Unlike NVivo 12 [14], paper labels provided the necessary visual and textual components needed to allow a larger number of researchers to work together during in‐person analysis. For instance, labels included a title and a short summary explaining the specific findings and contributing studies. All labels were color‐coded according to health condition and whose interpretation was presented (i.e., that of the primary study author or the review team). Paper labels were also numbered to match the structure of an accompanying detailed Word [35] document which gave the full details of the primary study data underpinning the short summary of the findings. This strategy of visually displaying all findings helped the review authors to iteratively test different ways of thematically grouping the findings. It also helped to conduct a thematic analysis with the creation of new themes signposted using Post‐it notes. Photos were taken to record different versions (e.g., version 1, version 2, etc.) so that the review authors could follow the development of their analysis and subsequent synthesis.

The use of paper labels supported the development of new themes and an initial draft of thematic groupings. Labels helped the review authors to efficiently analyze a large volume of rich data and findings as a team. Paper labels enabled teamwork in the identification of overarching concepts and creation of new understandings or concepts during a synthesis meeting.

**Figure 3:**
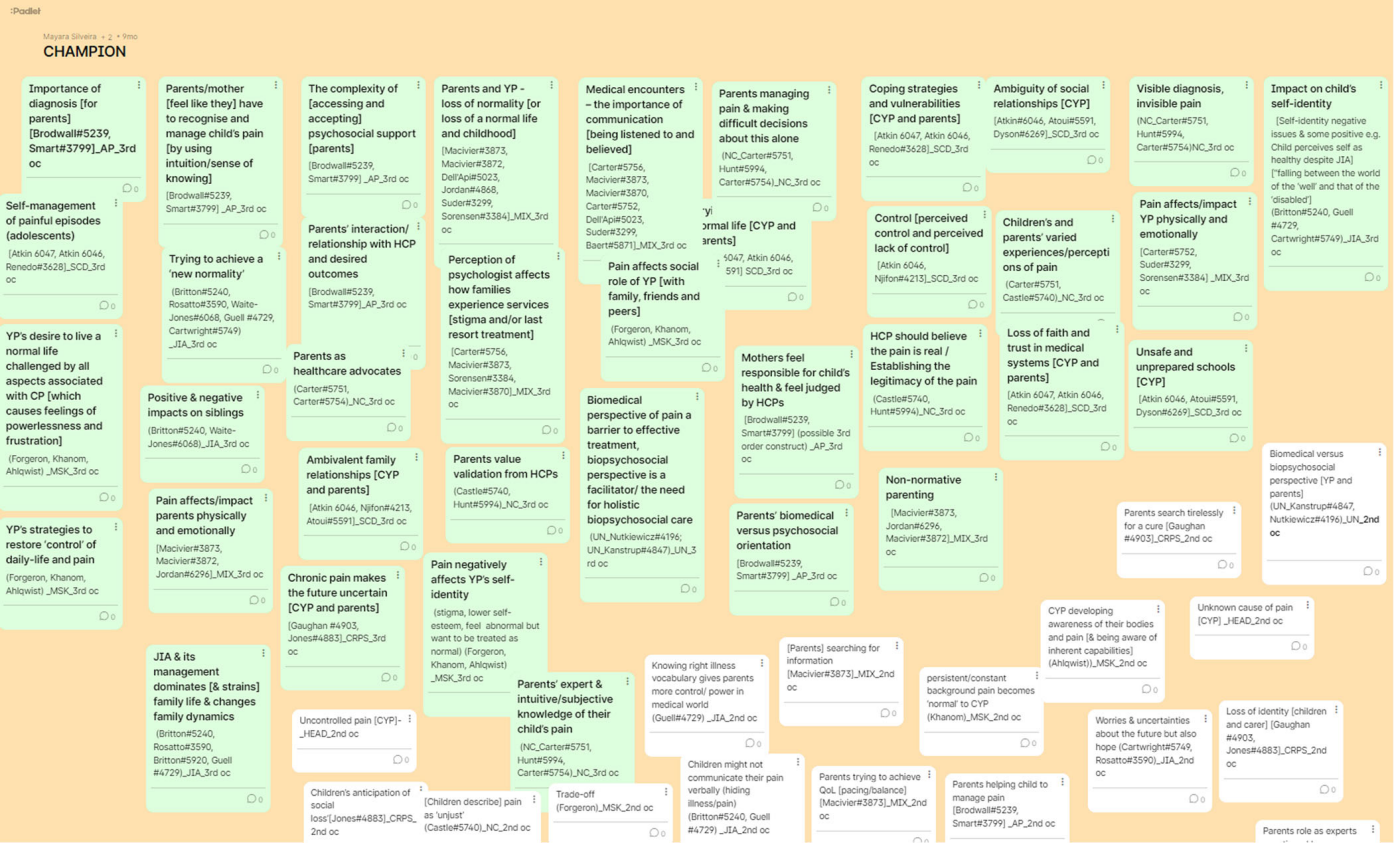
Virtual labels created on Padlet [11] to allow online collaboration on data analysis during a team meeting.

When working remotely, the review authors adapted the method by recreating all paper labels virtually using Padlet [11] (Figure 3), a real‐time collaborative web platform. Padlet [11] virtual labels were color‐coded according to whose interpretation was presented (i.e., whether it was the interpretation of the primary study author or of the review team) and included a title and description of the construct. Both physical (Figure 2) and virtual labels were used together during the team meeting. The idea was that members joining remotely via Microsoft Teams [19] could participate in the thematic synthesis in real time using the virtual labels. However, the review authors learned that constantly updating Padlet [11] to match the thematic groupings in real time was challenging and time‐consuming. This process could have been more efficient with the involvement of a dedicated facilitator, who could have taken responsibility for regularly updating the Padlet [11]. As a result, the review authors that were joining the meeting online were updated verbally regarding the changes in the configurations of labels and Padlet was used only as a visual aid. At the end of the meeting, photos showing the labels that were used to create “new constructs” or understandings were uploaded on Padlet [11] to facilitate discussion with the whole team and to provide a record of the analysis (Appendix S1).

### 1.7 Interactive whiteboard—Google Jamboard

**Figure 4:**
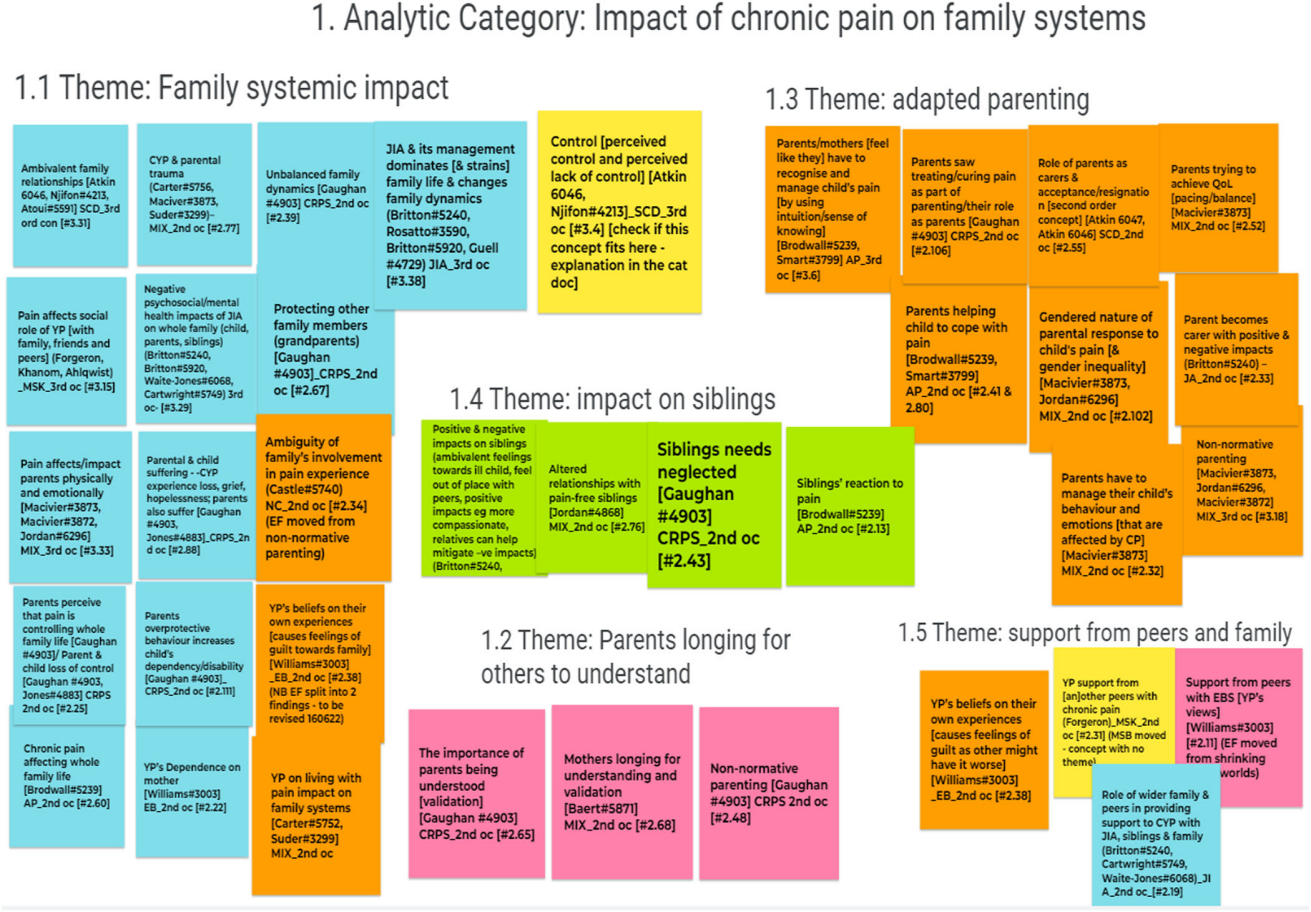
Virtual Post‐it notes on Google Jamboard to allow online collaborative data analysis during a team meeting.

A digital interactive whiteboard, Google Jamboard, was used during phase 6 of the meta‐ethnography to display data and develop analytic categories remotely [21]. Google Jamboard [21] is composed of different “frames,” similar to pages or slides. The authors used each frame to analyze a specific cluster of related themes, which were grouped together into a broader “analytic category,” for instance, as shown in the frame in Figure 4. All findings were recreated as notes that were color‐coded according to the “analytic category” to which they belonged. All notes included a title, the contributing studies, the health condition, and whose interpretation was presented (i.e., that of the primary study authors or the review team). The “analytic categories,” themes, and their constituent findings and all notes were numbered to match the same structure as the textual synthesis (i.e., a Word [35] document containing the full details of the primary study data underpinning the findings, themes, and analytic categories). This strategy allowed the authors to easily transfer any changes or new interpretations into the textual synthesis document. Google Jamboard [21] also facilitated the tracking of how the themes were organized according to the different interpretations from the team and facilitated team discussions of the different interpretations.

Using Google Jamboard [21] to display the analytic categories, themes, and their constituent findings resulted in the creation of five analytic categories that organized the whole textual synthesis. This visual method was crucial to allow interactive online analytic synthesis meetings using all the different perspectives and expertise from the whole research team (Figure 4).

### 1.8 Interactive whiteboard – Microsoft Whiteboard

This method was used during phase 6 of the meta‐ethnography to visualize and further develop the synthesis. Microsoft Whiteboard [19] is a multiplatform application which simulates a virtual whiteboard and enables real‐time collaboration. The review authors used Microsoft Whiteboard [19] to express and understand how findings were connected to one another to create a coherent “storyline” [line of argument] (see Section 1.3). Initially, the authors included all themes under their respective category as text boxes on Whiteboard [19]. All text boxes were color‐coded according to context (i.e., different colors were used to indicate starting points, potential links with other categories, and findings representing a positive impact). The authors used arrows to indicate which findings/themes were related, and the result was a large diagram linking all five categories (Appendix S2). Short descriptions for each analytic category were created based on the diagrams and these were discussed during an analysis meeting with the research team. At this point, the review authors focused on creating a better understanding of each analytic category. Subsequently, the diagram was further developed incorporating different interpretations and perceptions from the multidisciplinary team, resulting in major modifications to allow a more in‐depth exploration of these data (Appendix S3).

**Figure 5:**
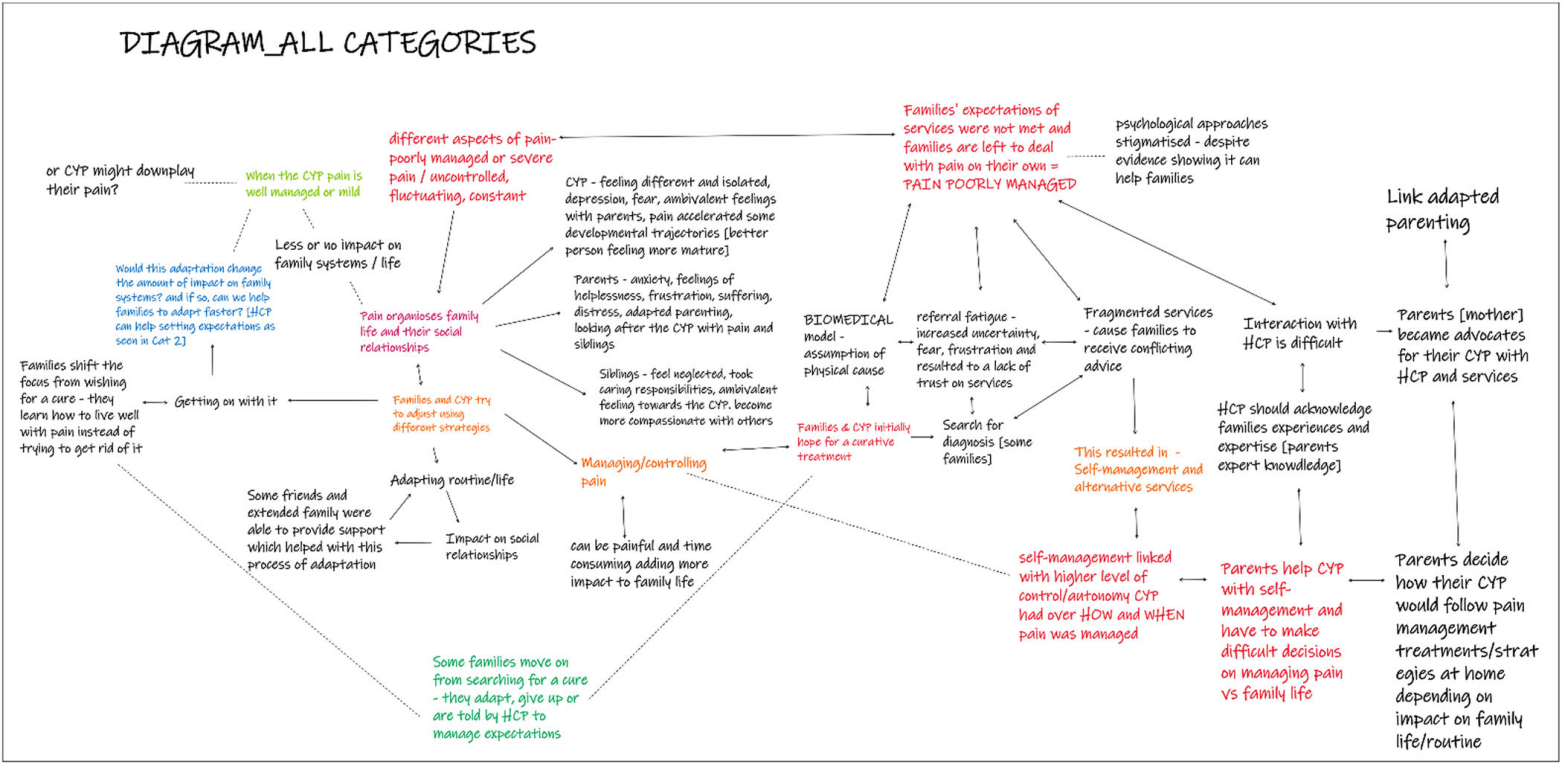
Final diagram developed from the process of interpreting findings using MS Whiteboard [19].

At this stage, the visual representation of all analytic categories in the form of diagrams allowed the team to develop their understanding of and start developing the initial “overarching storylines” or lines of argument. The initial interpretations and hypotheses were inserted in the diagram as virtual notes. The final step was the creation of a further simplified version of the diagram (Figure 5) that displayed how all four final analytic categories and findings were connected. The researchers used this last version of the diagram to further develop the description of the diagram to include how all categories and themes/findings were related which was used to create the textual synthesis.

Microsoft Whiteboard [19] was used to develop the overarching storylines which culminated in the development of three lines of argument. This process also resulted in the development of four analytic categories and the initial textual synthesis. While the Whiteboard [19] allowed real‐time collaboration and facilitated teamwork, it only worked well with the core research team of two people as it was hard for the wider team to keep track of or readily interpret the large and complex diagrams.

### 1.9 Cartoons and infographic

**Figure 6:**
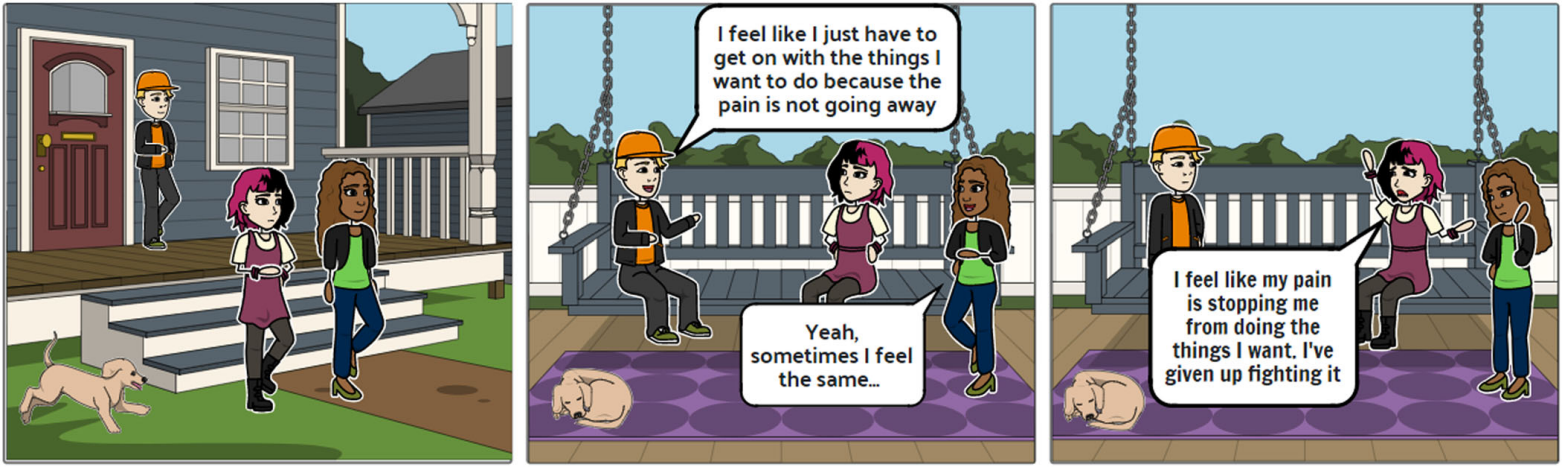
Cartoon—title: Different ways of thinking about pain. Aaron invited his friends Louise and Chloe for a catch‐up in his house. They all have chronic pain.

Cartoons and an infographic were used during phase 6 (synthesizing translations) of the meta‐ethnography to engage stakeholders and further develop and clarify the synthesis findings. PPI was fundamental to help clarify ambiguous or unclear findings. The review authors delivered virtual workshops with parents and young people to discuss, clarify, and interpret preliminary findings of the synthesis. Storyboard [36] was used to create cartoons (Figure 6) to convey ambiguous and unclear findings to prompt discussion among the PPI members and a scenario was created for each cartoon to facilitate this process.

**Figure 7:**
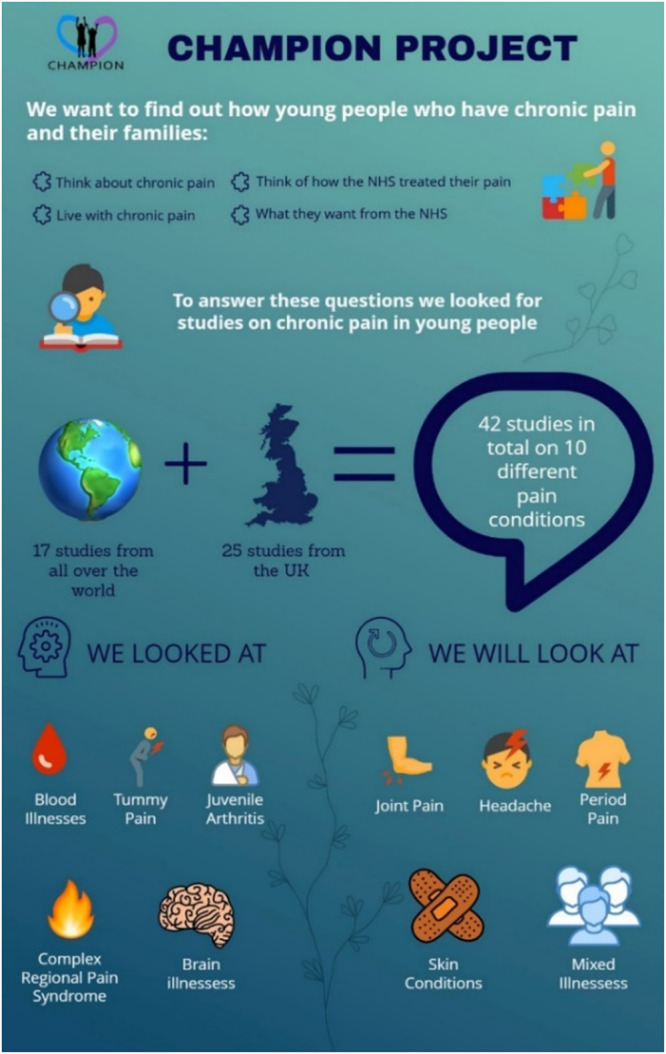
Infographic summarizing study progress.

The cartoons were representative, including people of different ethnicities and genders and the language used was accessible and engaging for children around 8–9 years of age. Patient and public members received the cartoons along with an infographic (Figure 7) explaining the preliminary findings a week prior to the workshop. The use of cartoons and an infographic to engage PPI members in facilitated discussions on what some concepts and findings would mean to parents and young people, providing context and adding nuances based on lived experience to some of the findings. Subsequently, new data and insights were incorporated into the analytic synthesis and were used to further refine and develop the interpretation of findings.

### 1.10 Diagrams to express the synthesis findings

**Figure 8:**
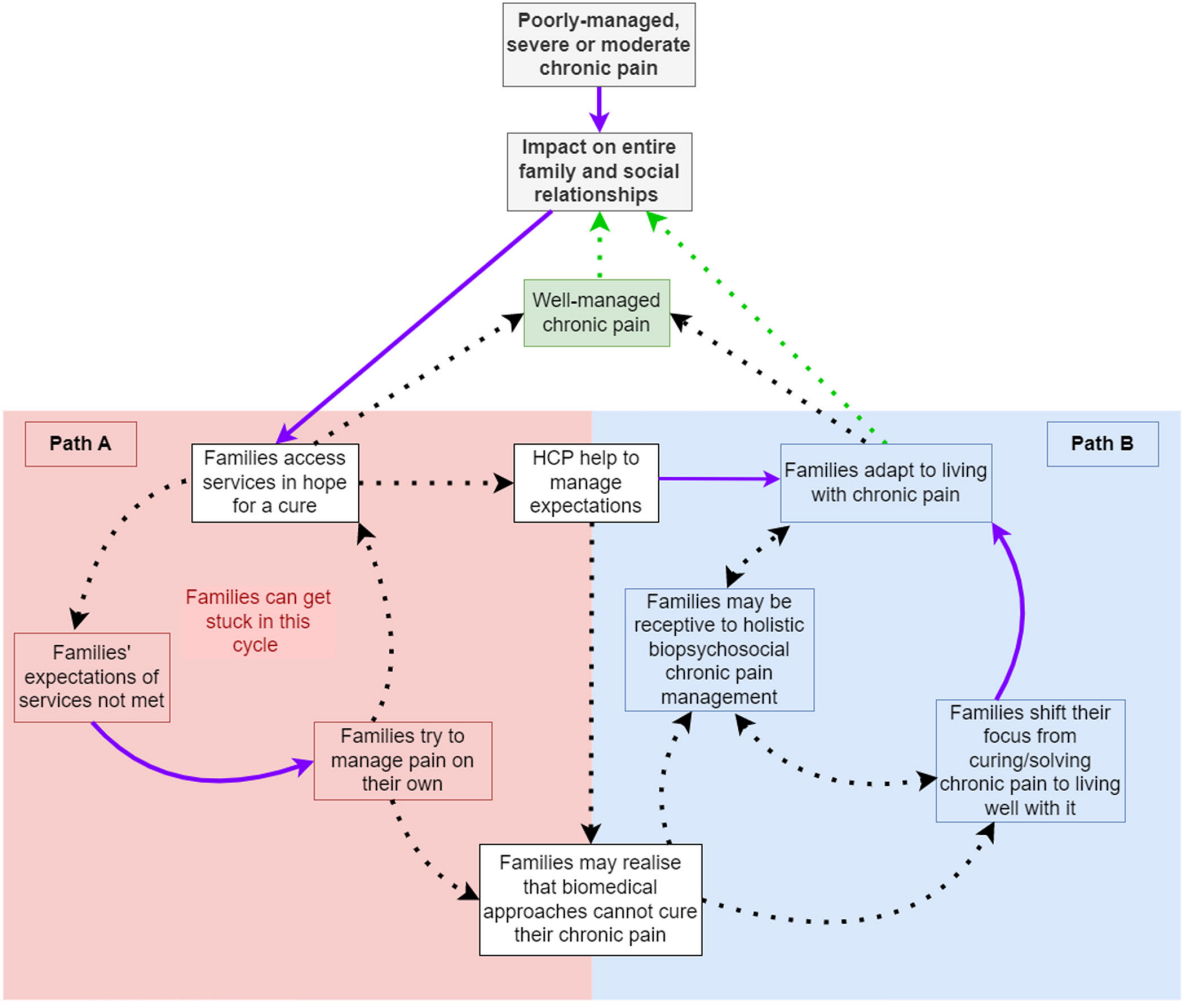
Model expressing the connections between all three lines of argument developed by France et al. [12, 13]. HCP, healthcare professionals.Key: 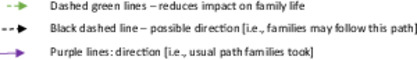

Diagrams were used by the review authors during phases 6 and 7 to further understand and express how the three lines of argument they developed were related. They used the final diagram they had produced using Microsoft Whiteboard [19] (Figure 5), data from the PPI workshop, and the textual synthesis, to create an initial version of a visual model to refine and represent the findings of the synthesis connecting all lines of argument in Microsoft Word [35]. The initial synthesis model was developed following feedback from the whole research team and depicted the nonlinear nature of the phenomenon of interest (i.e., families' journeys living with chronic pain and how they are affected by services). Subsequently, the researchers used Drawio [37] to draw and refine the model with the inclusion of more context and nuance. This process of further refining the model consisted of rich interpretative discussions among the core members of the research team until an intuitive final version was constructed (Figure 8). The synthesis model expressed the concept of a journey families are navigating while they deal with chronic pain and access services. To express the concept of the journey and time, the researchers used rounded arrows to create an illusion of a cycle and described (text in red) where families might stay “stuck.” Two text boxes between both pathways indicated how families might navigate between these distinct pathways.

The output was the final development and expression of the synthesis with a model produced initially in Microsoft Word [35] and finalized in Drawio [37] The model was fundamental to finalizing the synthesis, as it allowed remote teamwork and the incorporation of nuances and context provided during the PPI workshop. The model also enabled the expression of the overarching storyline connecting all lines of argument and the visualization of a complex nonlinear phenomenon.

### 1.11 Diagrams to develop and express theory

**Figure 9:**
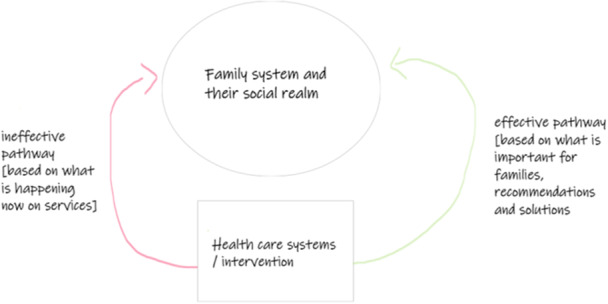
Conceptualization of the theory of good chronic pain management.

Diagrams were used during phases 6 and 7—synthesizing the translations and expressing the synthesis. The review authors produced a theory explaining their phenomenon of interest (i.e., theory of good chronic pain management). This process included multiple analysis meetings with the core review authors and it also integrated insights from PPI lived experience and the key findings from the synthesis of the studies included in the review data. The authors used two of their main analytic categories (related to family life and their social relationships and their experiences navigating health services) to construct the initial structure for the theory in the center of the diagram on Microsoft Whiteboard [19]. The review authors then placed all factors that had a positive impact on family life on the right side of the diagram, and factors with a negative impact on the left side. They used arrows to indicate when an aspect could be modified by the factors placed on each side of the diagram. The final version of the diagram mapped all factors that had the potential to “modulate” families' experiences with chronic pain (Appendix S4). Figure 9 shows the simplified version of the diagram.

**Figure 10:**
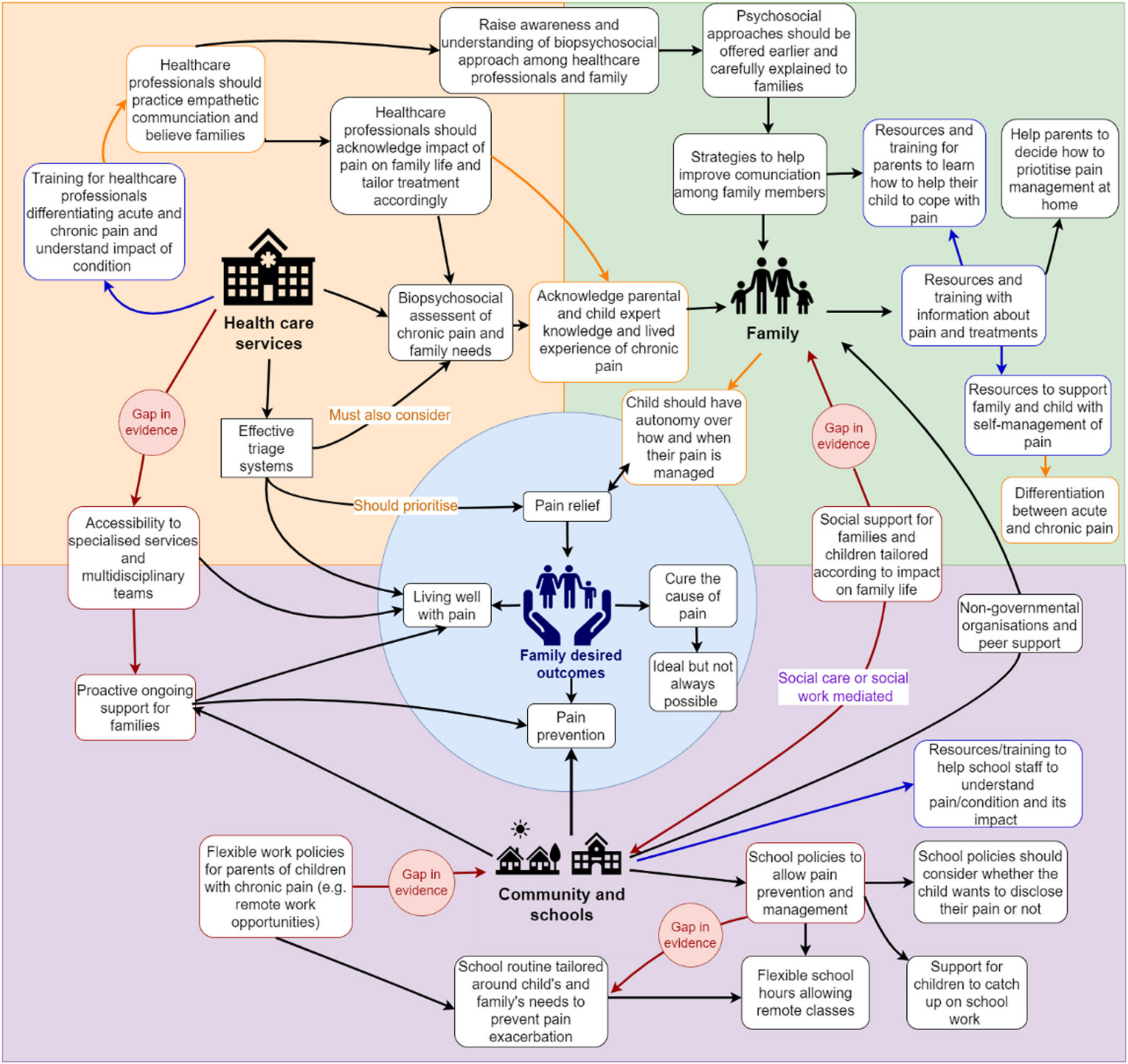
Diagram depicting theory of good chronic pain management developed by France et al. [12, 13]Key: 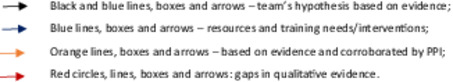.

The factors that positively impacted family life and their experiences with services were then developed into actions in a whole systems biopsychosocial theory. This was achieved with further interpretation of the key findings (i.e., key outcomes families consider as important) while drawing from expertise from the research team and PPI lived experiences. The Drawio [37] software was used to continue developing the theory, as it enabled clear visualization of the processes and facilitated discussions with the core research team. The final product expressed the whole system approach underpinning the theory through different background colors indicating different environments within the system (Figure 10).

The output was the conceptualization and expression of a theory produced initially with MS Whiteboard [19] and finalized in Drawio [37]. The diagrams were fundamental to developing the theory as they allowed remote teamwork and the mapping of all factors modulating the phenomenon of interest. The expression of the theory through the diagram also allowed a clear visualization of gaps in the data, and where the evidence was based on lived experiences or on the research team's hypothesis. The use of the diagrams enabled complex analysis and supported the convergence of evidence from different sources into a detailed theory. More examples illustrating the use of diagrams in QES are available in Table 1.

## 2 DISCUSSION

We have shown that the use of additional visual methods in a QES facilitated better data visualization, remote analysis group meetings, interpretation of large amounts of data, and meaningful PPI during synthesis. Visual methods varied in complexity, costs, and required expertise, allowing flexibility to adapt to different contexts, whether virtual or face‐to‐face. For instance, certain methods such as paper labels worked better in face‐to‐face settings and facilitated group work involving multiple people. This method was essential to allow group work when dealing with large amounts of data. In contrast, the use of labels in virtual platforms such as Padlet [11] was time‐consuming and demanded the presence of a facilitator and could only cope with moderate amounts of data.

Virtual platforms for implementing virtual methods remotely also worked differently depending on the task. For example, whilst Google Jamboard [21] facilitated collaboration with the wider team as it was more accessible and interactive compared to the textual synthesis, it did not allow the analysis of a large amount of data. Each frame could only cope with one main analytic category and required a facilitator to enable discussion. In contrast, Microsoft Whiteboard [19] allowed the processing of a large amount of data but only the collaboration of a small team of two people. Irrespectively, both methods allowed interactive online analytic synthesis meetings in different phases of the QES and were crucial for the development of findings.

It is imperative to carefully consider equity, inclusion, and diversity in the development and application of visual methods to ensure their accessibility and relevance across diverse populations. In addition, when developing and tailoring all virtual methods or outputs, the review authors need to carefully consider their personal biases and professional perspectives and positioning concerning what they would choose to present visually and how they interpret it. As such, it is essential that visualizations are either informed by or created with those they represent so they are inclusive and relatable to their audience. For example, in France's meta‐ethnography [12, 13] the authors co‐developed cartoons with members of the public and were careful to ensure these were representative of different ethnicities, and genders and did not promote an idealized context. The review authors also carefully considered the scenario and context of each cartoon ensuring these were appropriate and inclusive (e.g., a plain doctor's surgery and non‐descript hospital settings). Visual methods should also use accessible and engaging language and include accessibility features such as subtitles, image, and audio descriptions.

## 3 CONCLUSIONS

To the best of our knowledge, this is the first study to date showing the application of additional visual methods in a published QES. Visual methods are currently underused and underreported in QESs. QES authors should consider making use of available visual methods, particularly when involving members of the public during synthesis. Selecting the appropriate visual method for synthesis should be guided by its ability to enhance analysis and align with the study's objectives. Methods must suit the content and stage of synthesis, whether for idea development or presenting findings. Practical factors, including available resources and the team's technical skills, are crucial, especially for remote work. Additionally, group‐based methods may require significant preparation, facilitation, and specialized skills. Careful consideration of these aspects will ensure the effective and efficient use of visual methods.

## AUTHOR CONTRIBUTIONS

Dr. Mayara Silveira Bianchim, Professor Emma France and Professor Jane Noyes all participated in conceptualization, data curation, formal analysis, investigation, methodology, project administration, resources, software, visualization, writing of original draft and review and editing.

## CONFLICT OF INTEREST STATEMENT

The authors declare no conflict of interest.

### PEER REVIEW

The peer review history for this article is available at https://www.webofscience.com/api/gateway/wos/peer-review/10.1002/cesm.70009.

## Supporting information

Supporting Information.

## Data Availability

The authors have nothing to report.
