# Supplementary material for: Using visual methods to further enhance qualitative evidence synthesis
Source: Cochrane Evid Synth Methods. 2024 Dec 22;2(12):e70009. doi: 10.1002/cesm.70009 (PMC11698405; doi:10.1002/cesm.70009)
Supplement: Supplementary file 1 — Supporting Information. [file CESM-2-e70009-s001.docx]

Appendices

Appendix 1 - Paper Labels


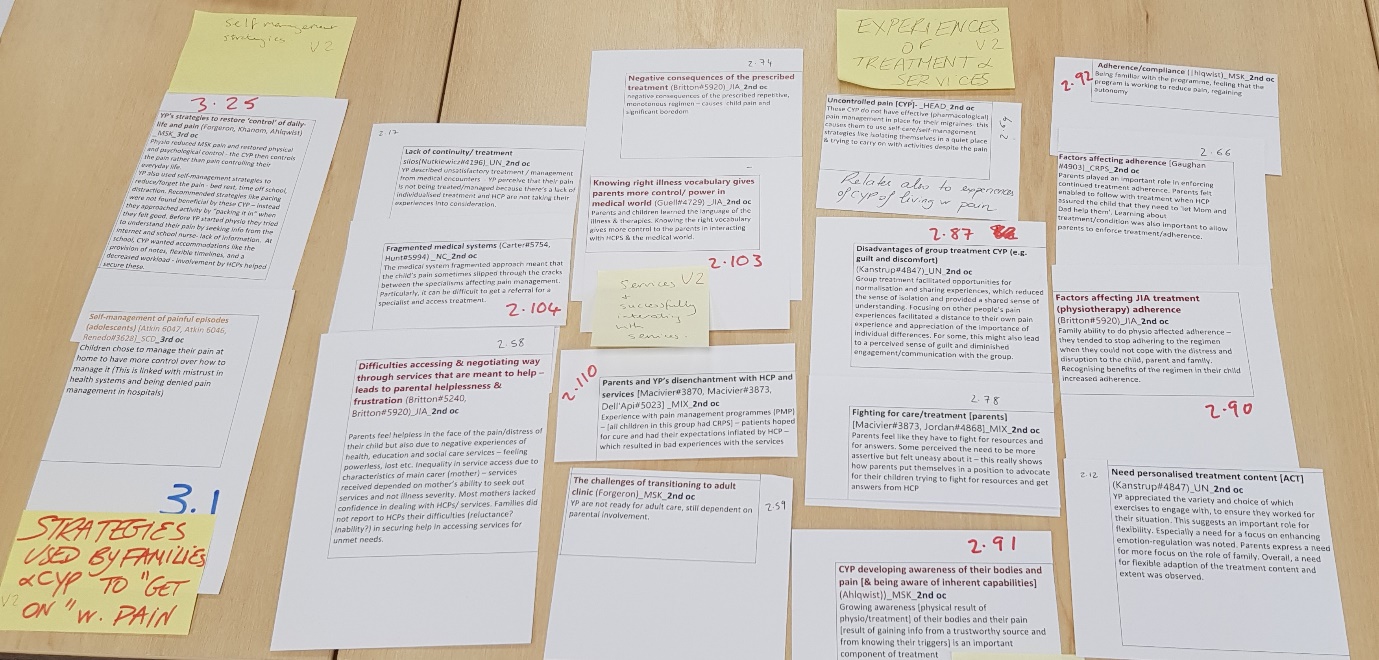


Figure A1. Paper labels used during team meetings

Appendix 2 – Short version of diagram


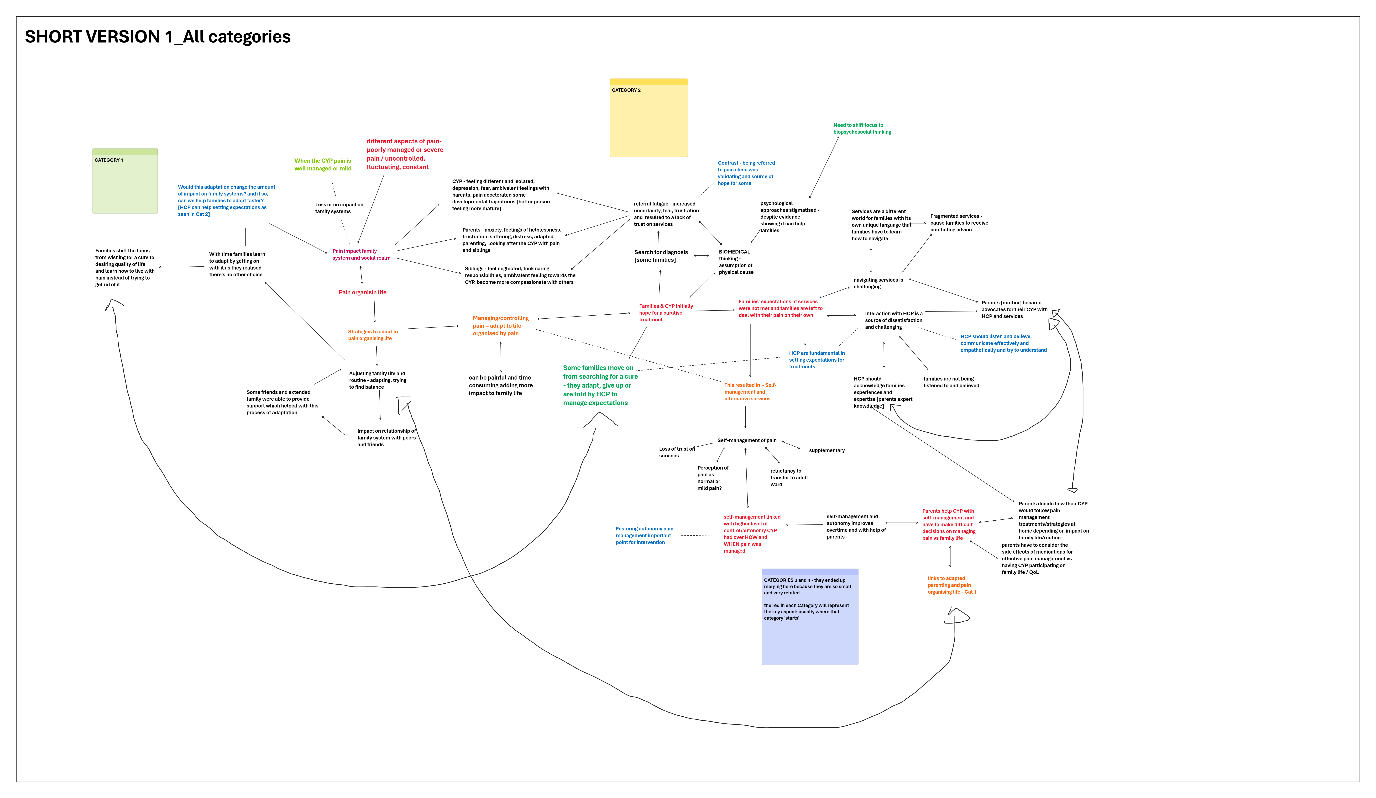


Figure A2. Short version of diagram created using Microsoft Teams

Appendix 3 – Long version of diagram


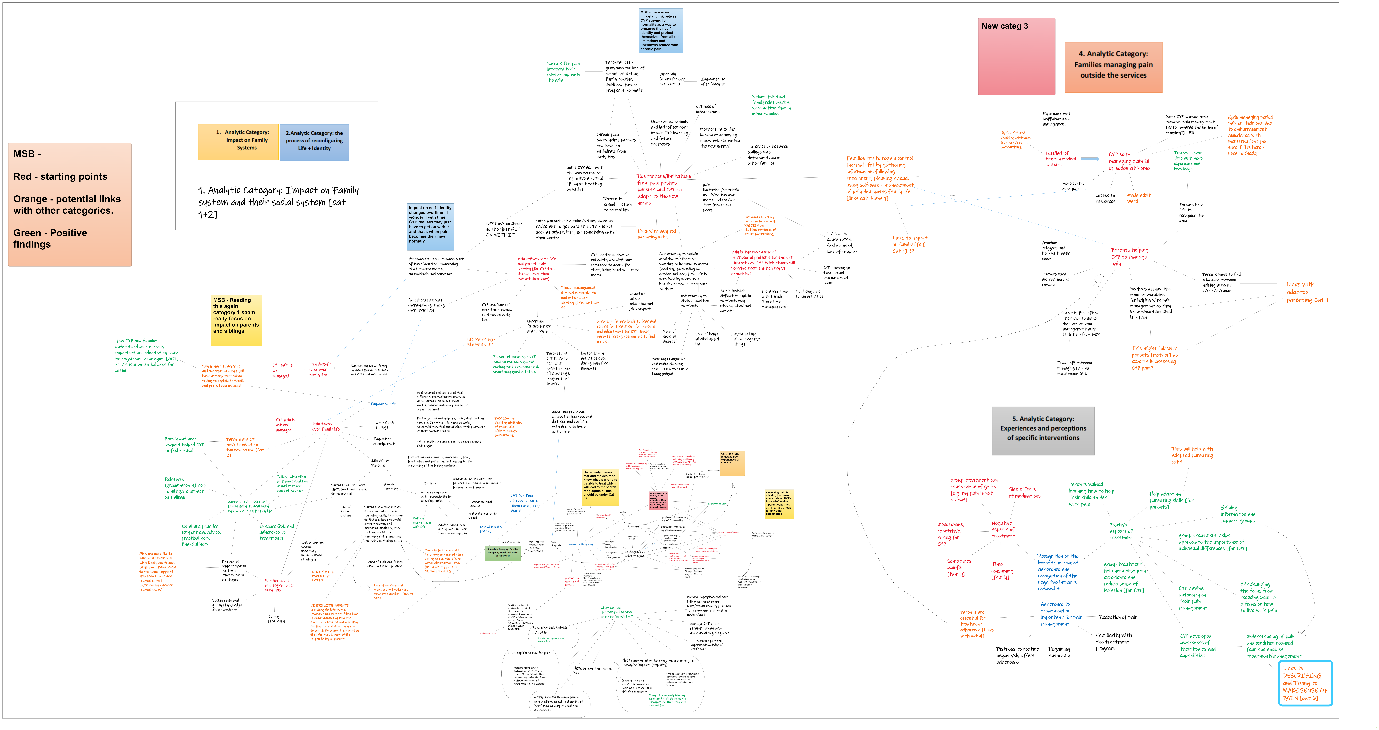


Figure A3. Long version of diagram created using Microsoft Teams

Appendix 4 – Detailed version of final diagram


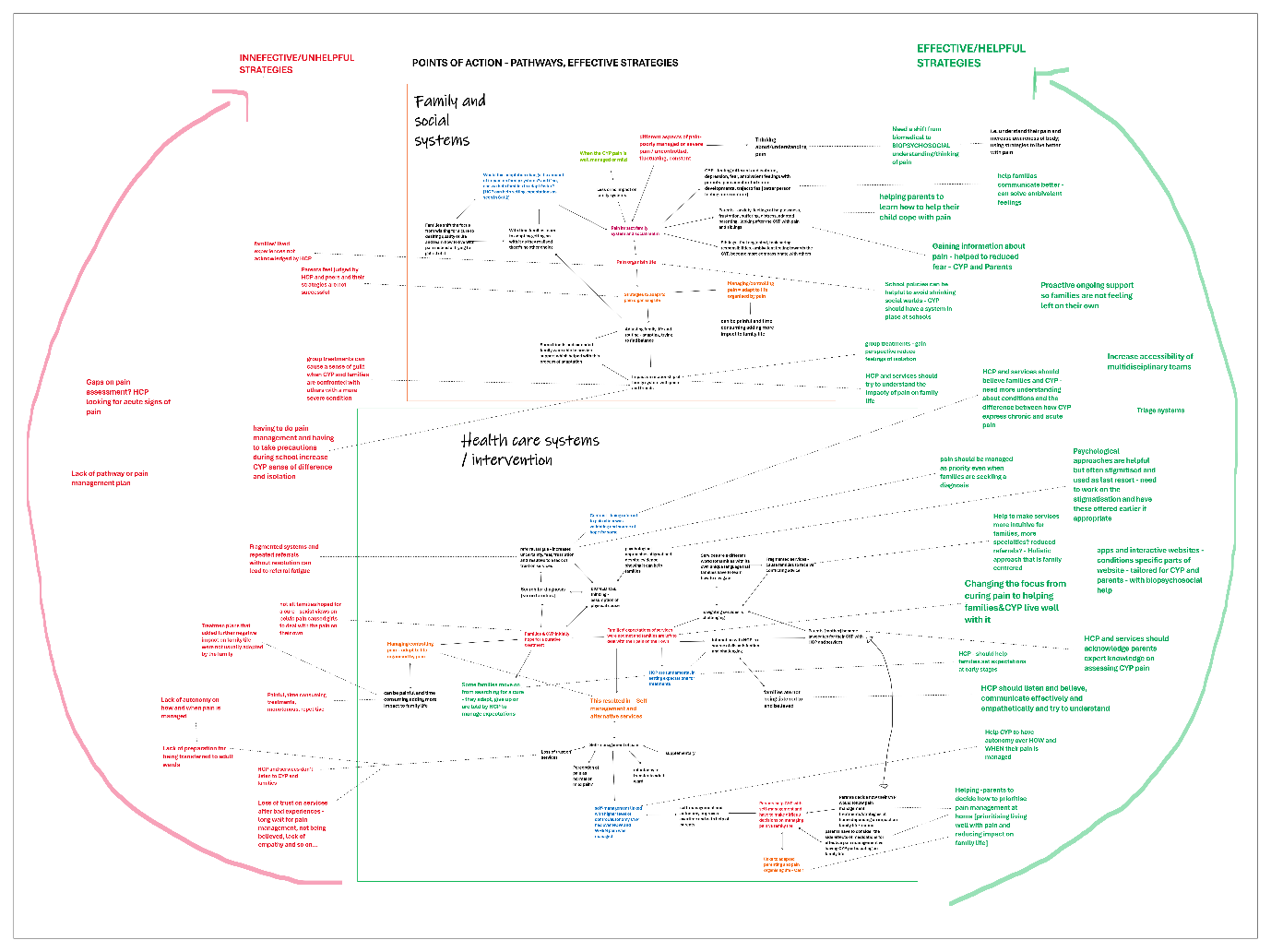


Figure A4. Detailed version of final version of the diagram created using Microsoft Whiteboard
